# Andrographolide suppresses hypoxia-induced embryonic hyaloid vascular system development through HIF-1a/VEGFR2 signaling pathway

**DOI:** 10.3389/fcvm.2023.1090938

**Published:** 2023-02-08

**Authors:** Zhong Jin, Qiru Guo, Zheng Wang, Xiao Wu, Wangming Hu, Jiali Li, Hongfei Li, Song Zhu, Haidi Zhang, Zixian Chen, Huan Xu, Liangqin Shi, Lan Yang, Yong Wang

**Affiliations:** ^1^College of Basic Medicine, Chengdu University of Traditional Chinese Medicine, Chengdu, China; ^2^Chengdu University of Traditional Chinese Medicine, Hospital of Chengdu University of Traditional Chinese Medicine, Chengdu, China; ^3^School of Ethnic Medicine, Chengdu University of Traditional Chinese Medicine, Chengdu, China

**Keywords:** andrographolide, hypoxia, HIF-1a, VEGF, embryonic hyaloid vasculature development

## Abstract

**Introduction:**

Ocular abnormalities and the development of retinal vasculature may cause postnatal retinopathy. In the past decade, tremendous progress has been made in identifying the mechanisms that regulate retina vasculature. However, the means of regulating embryonic hyaloid vasculature development is largely unknown. This study aims to determine whether and how andrographolide regulates embryonic hyaloid vasculature development.

**Methods:**

Murine embryonic retinas were used in this study. Whole mount isolectin B4 (IB4) staining, hematoxylin and eosin (H&E) staining, immunohistochemistry (IHC), and immunofluorescence staining (IF) were performed to determine whether andrographolide is critical for embryonic hyaloid vasculature development. BrdU incorporation assay, Boyden chamber migration assay, spheroid sprouting assay, and Matrigel-based tube formation assay were performed to evaluate whether andrographolide regulates the proliferation and migration of vascular endothelial cells. Molecular docking simulation and Co-immunoprecipitation assay were used to observe protein interaction.

**Results:**

Hypoxia conditions exist in murine embryonic retinas. Hypoxia induces HIF-1a expression; high-expressed HIF-1a interacts with VEGFR2, resulting in the activation of the VEGF signaling pathway. Andrographolide suppresses hypoxia-induced HIF-1a expression and, at least in part, interrupts the interaction between HIF-1a and VEGFR2, causing inhibiting endothelial proliferation and migration, eventually inhibiting embryonic hyaloid vasculature development.

**Conclusion:**

Our data demonstrated that andrographolide plays a critical role in regulating embryonic hyaloid vasculature development.

## Introduction

Abnormal development of the retina vasculature system may cause postnatal retinopathy such as retinopathy of prematurity, familial exudative vitreoretinopathy, diabetic retinopathy, and age-related macular degeneration (1). The choroidal vasculature network exists in the outer aspect of the retinal pigmented epithelium and provides oxygen and nutrition to the developing retina through diffusion (2), whereas metabolic supplement to the inner retina depends on the hyaloid vasculature. The vascular network of the inner portion of the retina undergoes remarkable changes and reorganization. The hyaloid vasculature, an arterial network, which arises from the optic disc, spreads forward dendritically, wraps around the outside of the lens, and exits through an annular collection vessel in front of the eye (2, 3). However, the hyaloid vessels regress when a vascular plexus emerges from the optic nerve head, which gives rise to retina vasculature. Unlike humans, mice and other rodents have an immature retinal vasculature and hyaloid vessels are persistent at birth (4). The primary retinal vasculature grows from the optic disc and spreads within the nerve fiber layer to form the superficial layer. Vessels grow from the primary vasculature vertically in the retina and two sets of capillary beds grow out from these vertically oriented vessels to form the deep layer and the intermediate layer (5, 6). The hyaloid vasculature is replaced by the retinal vasculature which contains both arteries and veins, and contributes to oxygen and nutrient supplement for the inner retina.

The development of the retina vasculature is closely associated with the oxygen supplement. The pathologic growth of new retinal vasculature ultimately results in bleeding, detachment, and scarring of the neural retina (7). Retinopathy, often referred to as retinal vascular disease, is any impairment of the retina which may cause vision impairment. Multiple stresses can cause postnatal retinopathy, such as diabetes, hypertension, ischemia, atherosclerosis, and oxidative damage. Appropriated proliferation, migration of endothelial cells, recruitment of smooth muscle and perivascular cells, and proper remodeling of extracellular matrix are particularly important for angiogenesis and maintaining the function of vascular vessels (8, 9).

The retinal vascular complex comprises a variety of cell types, including vascular endothelial cells, pericytes, microglia, neuron, glia, astrocytes, and immune cells (2, 3). Retinal vascular development is coordinately controlled by multiple signaling pathways. Development of the retina vasculature is mediated by the hypoxia-induced vascular endothelial growth factor expressed by neuroglia (10). ANGPTL4 is essential for sprouting angiogenesis in both developmental and pathological conditions (9). Polyunsaturated fatty acid-associated metabolic disorder contributes to retinopathy development (11). Notch signaling regulates endothelial tip cell sprouting, which plays a critical role in retinal vascular development (12). CCN1 regulates retinal angiogenesis through interacting with VEGF and Notch signaling pathways (13). TGF-β1 suppresses retinal endothelial cell proliferation under pathological conditions (14). Erythropoietin promotes retinal angiogenesis, which may be associated with oxidative stress (15). The adenosine metabolic system can modulate normal retinal vascularization (16). Nerve growth factors contribute to pathological retinal neovascularization (17).

Tremendous progress has been made in the past decade in identifying mechanisms that regulate angiogenesis. The most well-known is identified as the VEGF family. In mammals, the VEGF family consists of five members: VEGF-A, placenta growth factor (PGF), VEGF-B, VEGF-C, and VEGF-D (18). All VEGF family members stimulate cellular responses by binding to tyrosine kinase receptors (VEGFRs) on the cell surface, causing dimerization and becoming activated through transphosphorylation (19). VEGF, a secreted polypeptide, plays a pivotal role during both vasculogenesis and angiogenesis (20–22). The expression of VEGF is controlled by HIFs, the intracellular oxygen sensors, which stabilize under tissue hypoxia conditions (22, 23).

The homeostasis of tissue microenvironments is indispensable in development. Interrupted vascular hemostasis, chronic inflammation, metabolism disorder, oldness, and underlying medical conditions are contributors to retinopathy development (24–26). Increased CO2 levels is associated with the inhibition of normal retinal vascular development and stimulates the peripheral avascular area in neonatal rats (27). Endothelial nitric oxide (NO) pathways regulate angiogenesis sprouting and pathological neovascularization through the regulation of endothelial cell polarity during retinopathy (28).

Ocular abnormalities and developing retinal angiogenesis may cause postnatal retinopathy. However, the means to regulate embryonic hyaloid vasculature development and the underlying mechanisms for regression of hyaloid vasculature are largely unknown. Andrographolide is the key compound isolated from *andrographis paniculate*. Andrographolide has beneficial effects for inflammatory diseases, such as virus infection, malaria, bacterial dysentery, fever, rheumatoid arthritis, laryngitis, and herpes. In recent years, novel pharmacological functions of andrographolide have been discovered by scientists, including inhibitory activity against pathogen-induced inflammation, treatment of colitis, and cardiovascular protection (29). Our previous studies demonstrated that andrographolide is critical in maintaining gastric vascular homeostasis, as well as regulating pathological vascular remodeling (30, 31). In this study, we aimed to determine whether and how andrographolide regulates embryonic hyaloid vasculature development.

## Materials and methods

### Animal ethical approval

The animals used in this study were approved by the Experimental Animal Ethics Committee of Chengdu University of Traditional Chinese Medicine.

### Animal treatment

Embryonic day was defined based on vaginal plug. C57/BL6 mice aged from 6 to 8 weeks were used in this study. The breeding cages were set up between 6:00–8:00 in the evening, and vaginal plugs were checked before 10:00 the next morning. At noon when the vaginal plug was observed, the day was defined as embryonic day 0.5 (E 0.5). The E10.5 pregnant mice were administrated andrographolide (10 mg/kg) *via* consecutive intraperitoneal injection at noon every day until sacrificed (30). Pregnant mice were sacrificed in an airtight tank filled with carbon dioxide. The heartbeat of pregnant mice was checked before embryos were collected. Embryos were placed in the carbon dioxide airtight tank again and embryonic eyeballs were collected.

### Cell culture and cell treatment

The human umbilical vein endothelial cell line (HUVEC-C, ATCC CRL-1730) was cultured with F-12 K medium (ATCC® 30–2004™) containing 10% FBS. Normal primary human umbilical vein endothelial cells (HUVEC, ATCC PCS-100-010) were cultured with a vascular cell basal medium (ATCC, PCS-100-030) supplemented with endothelial Cell Growth Kit (ATCC, PCS-100-040).

### Whole mount isolectin B4 staining of murine embryonic retinas and crystalline lens

E16.5 and E18.5 eyeballs were collected and fixed with 4% paraformaldehyde (PFA) for 30–60 min. The retinas and crystalline lenses were dissected and permeabilized in cold methanol for 30 min. They were then washed with PBS and incubated with Perm/Block solution for 1 h, followed by isolectin B4 (IB4) staining (1:100) (32). Images were captured using confocal microscopy (LS510, Zeiss) after isolectin B4 incubation overnight at 4°C.

### Hematoxylin and eosin staining, immunohistochemistry and immunofluorescence staining

Harvested eyeballs were fixed with 4% paraformaldehyde overnight at 4°C. 5 μm-thick slides were collected after paraffin embedding. H&E staining was performed as in our previous publication (33). For IHC staining, the slides were deparaffinized and antigen retrieval was performed with citric acid treatment at 98°C for 5 to10 min. After antigenic unmasking, the slides were incubated with CD31 (Cell Signaling Technology, 1:200) (34), HIF-1α (Cell Signaling Technology, 1:200) (35), and VRGF Receptor 2 (Cell Signaling Technology, 1:200) (36) overnight at 4°C. This was followed by incubation with biotinylated secondary antibodies at room temperature for 1 h (Vector Laboratories), and incubation in ABC solution (Vector Laboratories) for 30 min at room temperature. The targets were visualized after DAB solution was added. For IF staining, the deparaffinized slides were permeabilized with PBS containing 0.25% Triton-X-100, blocked with 10% goat serum, and incubated with primary antibodies overnight at 4°C. This was followed by incubation with Alexa 594-conjugated or Alexa 488-conjugated dary antibodies at room temperature for 1 h. Nuclei were visualized with 4′, 6′-diamidino-2-phenylindole (DAPI) staining. For BrdU staining, DNA was denaturized using 2 N HCl, and followed by incubation with antibodies. Images were captured using confocal microscopy (LS510, Zeiss).

### CCK8 cell proliferation assay

3 × 10^3^ HUVECs or HUVEC-Cs were seeded in 96-well culture plates (in each well). Absorbance at 450 nm was evaluated using Cell Counting Kit-8 after andrographolide (5 μM) treatment for 24 h.

### BrdU incorporation assay

HUVEC-Cs were treated with andrographolide (5 μM), following BrdU labeling reagent labeled for 24 h. Immunofluorescence staining was performed to determine BrdU incorporated HUVEC-Cs.

### Cell-cycle analysis with flow cytometry

HUVEC-Cs were treated with andrographolide for 24 h, then fixed with a concentration of 75% cold ethanol. Cells were incubated with a propidium iodide reagent for 15 min at 37°C. The cell cycle was analyzed using the FACSVerseTM system (Becton Dickinson). The percentage of cells in each phase of the cell cycle was determined using CellQuest v3.3 software (BD Bioscience).

### Boyden chamber migration assay

1 × 10^6^ HUVEC-Cs were suspended in 100 ul FBS-free culture media and seeded into the Boyden Chamber (353,097, FALCON), which was set up with 24-well culture plates containing 500 ul complete culture medium (10% FBS) and 5 μM andrographolide. After incubation for 12 to 24 h, crystal violet staining was performed to visualize the migrated cells. Those cells were manually counted in five random microscopic fields.

### Spheroid sprouting assay

The spheroid sprouting assay was performed as described previously (37). Methylcellulose solution was prepared by dissolving 6 g methylcellulose (sigma) in 250 ml prewarmed serum-free medium, and 250 ml complete medium was added. Suspended cells were added to dissolved methylcellulose solution which was prepared with 10 ml methylcellulose solution and 40 ml complete medium to form spheres. Neutralized collagen solution was added to the 24-well culture plates and incubated at 37°C until the collagen solidified. The spheres were mixed with the dissolved collagen solution and transferred to the solidified collagen culture plates. The culture plates were solidified for 30 min at 37°C. Then, 200 ul of complete medium containing andrographolide was added and cultured overnight. The spheroid sprouting was visualized after calcein AM staining. Images were captured using confocal microscopes (Leica Microsystem CMS GmbH); the number of sprouts and the total sprout length of each sphere were analyzed using Image J software.

### Matrigel-based tube formation assay

HUVEC-Cs were treated with andrographolide overnight. Those cells were seeded in growth-factor-reduced Matrigel (BD Bioscience)-coated 24-well plates (8 × 10^4^ cells in each well). The endothelial tubule formation was monitored under microscope. Images of tubes were captured using an inverted immunofluorescence microscope after calcein AM staining. Cumulative tube numbers and tube length were quantified using Image J software.

### Quantitative real-time PCR analysis

Total RNA from HUVEC or HUVEC-C was extracted using TRIzol reagent. Quantification of RNA was monitored by spectrophotometer (Denovix, USA). As a template, 600 ng RNA was used, and random hexamer primers were used for reverse transcription reaction to obtain cDNA using the iScript cDNA synthesis kit. Real-time PCR was performed in duplicate for each sample on the Bio-Rad Real-time PCR system. The primer sequences used in this study are exhibited in the supplementary data (Supplementary Figure 18). The relative gene expression level was analyzed using the 2^-ΔΔct^ method against β-Actin.

### Protein extraction and Western blotting

Protein from HUVEC-C was extracted using RIPA lysis buffer. Protein concentration was evaluated *via* BCA kit (Biosharp). Protein was denatured at 98°C for 10 min, then separated by sodium dodecyl sulfate-polyacrylamide gel electrophoresis (SDS-PAGE) and transferred onto polyvinylidene fluoride (PVDF) membranes. Afterwards, they were blocked with 5% fat free milk and incubated with specific antibodies at 4°C overnight. Images were captured using ImageQuant LAS 4000 Image Station, and ImageQuant TL software was used to quantify the densities of protein bands.

### Molecular docking simulation of andrographolide with HIF-1a and VEGFR2

A molecular docking simulation was performed to determine the binding energy of andrographolide with HIF-1a and VEGFR2 using Autodock Vina 1.5.6 software developed by Olson’s research group (38). The three-dimensional structures of HIF-1a and VEGFR2 were obtained from the RCSBPDB database (http://www.rcsb.org/). When a binding energy value was less than zero, those proteins were considered spontaneously binding and interacting with each other. The lower the binding energy, the more stable the molecular conformation.

### Co-immunoprecipitation assay

Total protein from HUVEC-C was extracted by using RIPA buffer. Precleared cell lysate using Anti-species-specific IgG beads. The precleared cell lysate was incubated with HIF-1α (Cell Signaling Technology) (35) and VEGFR2 (Cell Signaling Technology) (36) for 1 h at 4°C. This was followed by incubation with pre-equilibrated Protein A/G agarose beads on a rocking platform overnight at 4°C. The co-immunoprecipitated targets were evaluated using western blotting.

### Statistics

Quantitative data are presented as mean ± SEM from biological triplicates. The statistical analysis was performed using GraphPad prism software. Normal distribution was evaluated using the Kolmogorov–Smirnov test, and the statistical comparisons between two groups were analyzed using two-tailed unpaired Student’s *t* test or one- or two-way of variance (ANOVA), followed by Bonferroni’s *post hoc* tests when appropriate. Two-sided *p* values were also quantified. *p < 0.05 was considered statistically significant.

## Results

### Andrographolide suppresses murine embryonic hyaloid vasculature development

Our previous publication indicated that andrographolide was critical in regulating pathological vascular remodeling. The parallel-arranged superficial layer, deep layer, and intermediate layer were exhibited within retinal vasculature, whereas only hyaloid vasculature existed in the inner retina during embryonic development (Supplementary Figure 1). Whether andrographolide can regulate hyaloid vasculature development is largely unknown.

**Figure 1:**
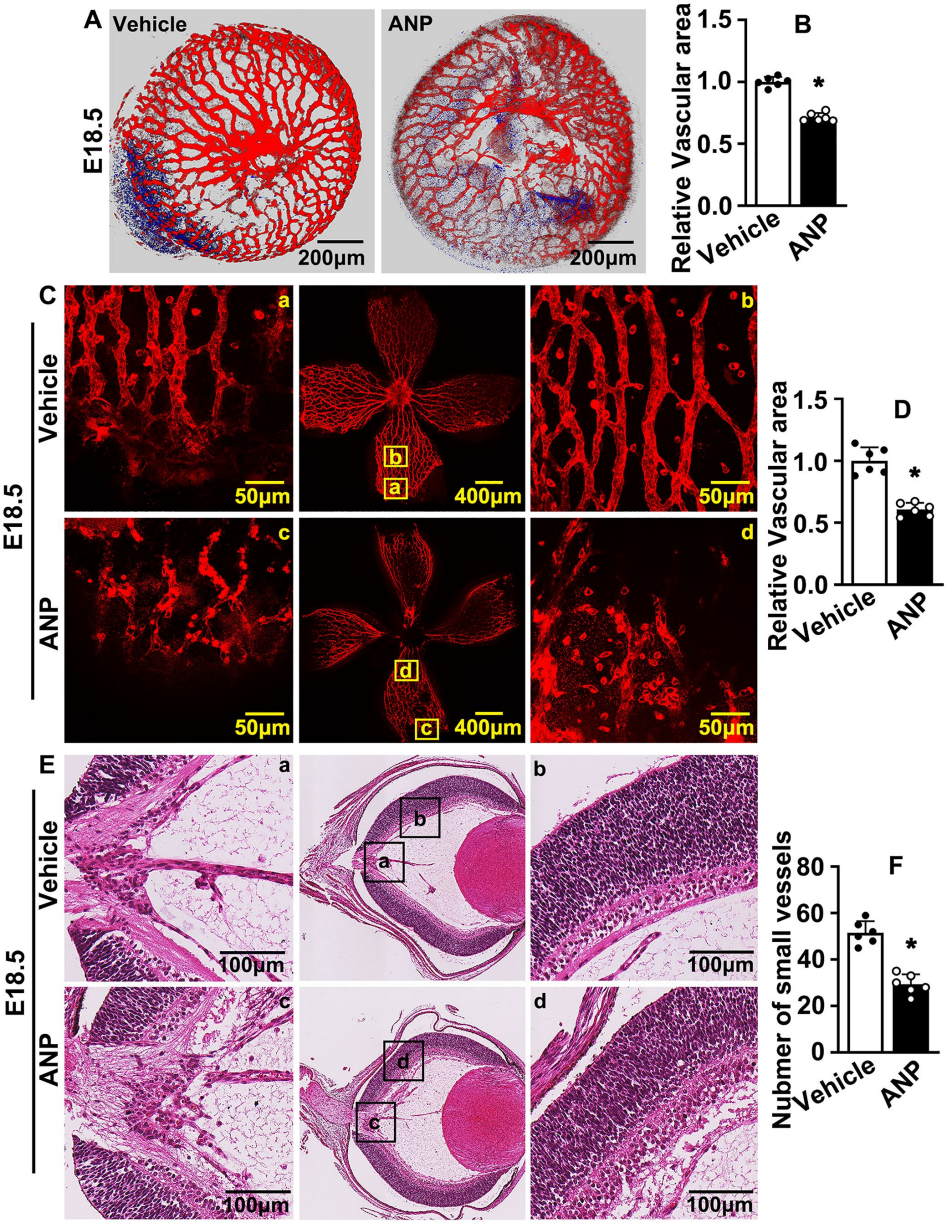
Andrographolide suppresses murine embryonic hyaloid vasculature development. **(A)** The E10.5 pregnant mice were administrated andrographolide (10 mg/kg) at noon everyday *via* consecutive intraperitoneal injection. Pregnant mice were sacrificed at E18.5. Retinas and crystalline lenses were isolated under microscope. Representative images of crystalline lenses at E18.5 with isolectin B4 (IB4) staining. Images were captured by 3D image stacks using confocal microscopy. **(B)** The quantification of the relative vascular area within crystalline lens at E18.5 (*n* = 6). **(C)** Representative images of retinas at E18.5 with IB4 staining. Whole images were exhibited in the middle sites and the high resolution images of the corresponding areas were displayed on both sides. **(D)** The quantification of relative vascular area of retinas at E18.5 (*n* = 6). **(E)** Representative images of H&E staining of eyeballs of E18.5 mice to exhibit hyaloid vascular vessels. Whole images were exhibited in the middle sites and the high resolution images of the corresponding areas were displayed on both sides. **(F)** Quantitative number of hyaloid vascular vessels at E18.5 (*n* = 6). Quantitative data are presented as mean ± SEM, *p < 0.05.

We checked the vaginal plug and consecutively administered andrographolide (10 mg/kg) by intraperitoneal injection until sacrificed (30). The retinas and crystalline lenses were collected and isolectin B4 staining was performed to visualize hyaloid vasculature development. The vascular network around the crystalline lens was well formed at E16.5. Andrographolide treatment attenuated the number of branched vessels, and more vascular plexuses characterized by red dot was localized tightly to existing (Supplementary Figures 2A,B). The smeared hyaloid vascular network was exhibited after andrographolide treatment, and the outline of vessels could not be distinguished (Supplementary Figure 3). The vascular network of the crystalline lens at E18.5 was determined by Z-sequences of optical slices using confocal microscopy. We found that the branched vessels, the diameter of formed vessels, and the connections between formed vessels in the crystalline lens were observably decreased following andrographolide treatment (Figures 1A,B). The hyaloid vascular network at this stage was also impaired, only large vessels could be distinguished with more vascular plexuses characterized by red dots among those smeared vascular networks being exhibited after andrographolide treatment (Figures 1C,D). We also performed H&E staining to visualize the structure of the retinal wall, and observed that the number of neurons and the thickness of retinal walls were not affected after andrographolide treatment. However, many more gaps around existing vessels were exhibited in the andrographolide treatment group (data not shown). We also quantified the hyaloid vascular endothelial cell nuclei to evaluate small vessel numbers (39), and our results indicated that the number of small vessels decreased after andrographolide treatment (Figures 1E,F). We further evaluated vessel formation with immunohistochemistry staining against CD31 antibodies on paraffin-embedded slides. Vessels within the optic disc were not affected after andrographolide treatment due to those large vessels being formed at early stages. However, the numbers of peripheral vessels and the integrated optical density (IOD) based on CD31 staining were dramatically decreased after andrographolide treatment (Supplementary Figures 4A–C). Our data demonstrated that andrographolide impaired murine hyaloid vasculature development.

### Andrographolide suppresses endothelial cell proliferation

**Figure 2:**
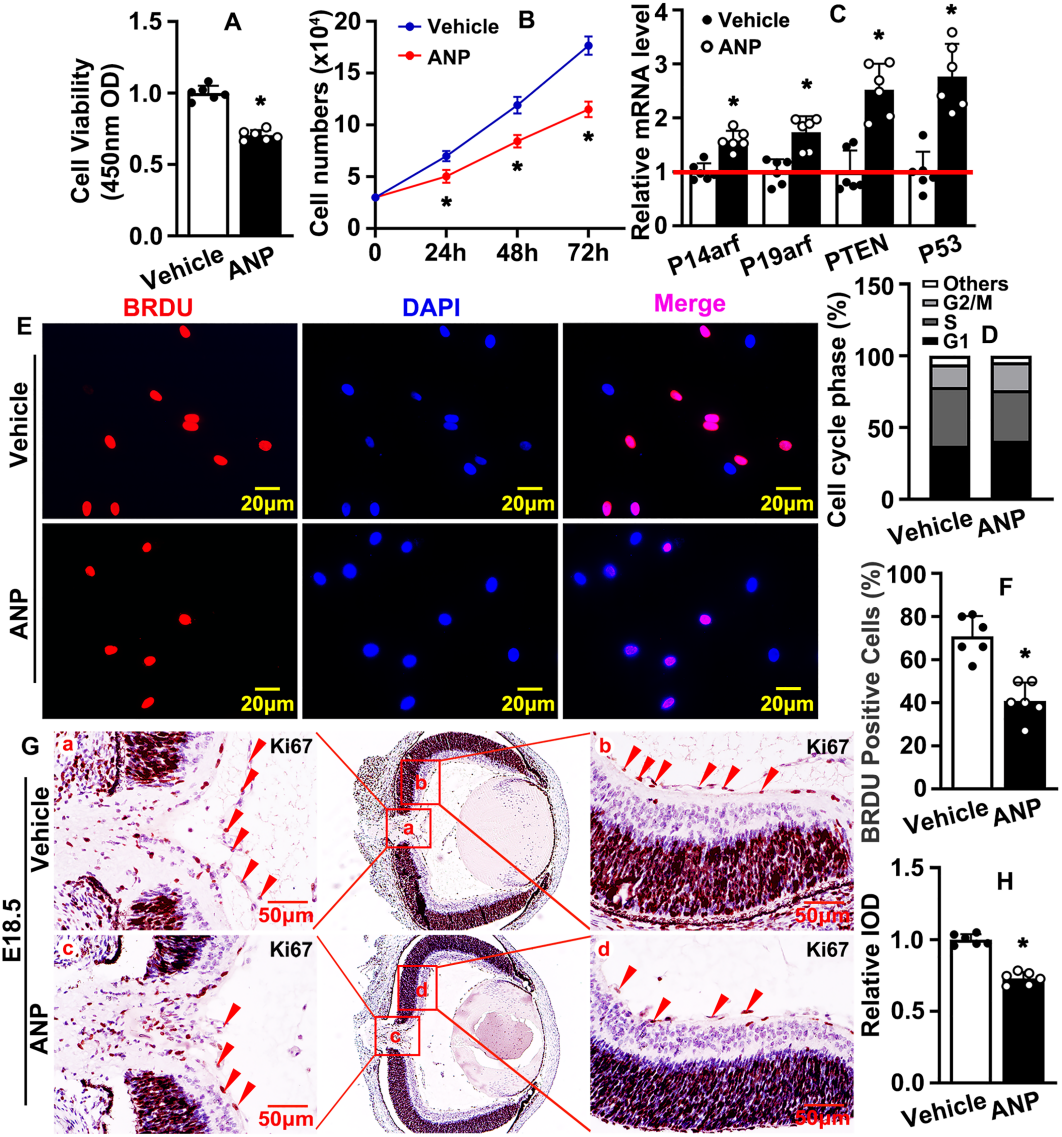
Andrographolide suppresses endothelial cell proliferation. **(A)** 3 × 10^3^ HUVECs were seeded in 96-well culture plates, absorbance at 450 nm was evaluated using Cell Counting Kit-8 after Andrographolide (5 μM) treatment for 24 h (*n* = 6). **(B)** HUVEC-Cs were treated with andrographolide (5 μM), the cell numbers were counted at different time points (24 h, 48 h, and 72 h) (*n* = 6). **(C)** HUVEC-Cs were treated with andrographolide (5 μM) and Real-time PCR was performed to determine cell cycle negative regulated genes (*n* = 6). **(D)** HUVEC-Cs were treated with andrographolide (5 μM) and cell cycle was determined by Flow Cytometer analysis after Propidium Iodide (PI) staining. The quantification of Flow Cytometry analysis of PI staining in HUVEC-Cs with andrographolide (5 μM) treatment for 24 h (*n* = *3*). **(E)** HUVEC-Cs were treated with andrographolide (5 μM) overnight, following BrdU labeling reagent labeled for 24 h. Immunofluorescence staining performed to determine BrdU-incorporated HUVEC-Cs. **(F)** Quantitative representation of BrdU positive cells in (E) (*n* = 6). **(G)** Immunohistochemistry (IHC) staining against Ki67 was performed to evaluate Ki67 expression in eyeballs at E18.5 after andrographolide treatment. **(H)** The relative expression of Ki67 was quantitated by integrated optical density (IOD) using Image J software (*n* = 6). Quantitative data are presented as mean ± SEM, *p < 0.05.

A variety of cell types contribute to retina vasculature development. In this study, we aimed to determine whether andrographolide regulated the vascular endothelial cell-mediated hyaloid vasculature. The proliferation of endothelial cells play a pivotal role during angiogenesis. To determine whether Andrographolide suppressed the proliferation of endothelial cells, we treated HUVEC with 5 μM andrographolide, and a Cell Counting Kit-8 assay was performed to evaluate cell viability and proliferation. We observed that andrographolide treatment significantly suppressed absorbance (OD) at 450 nm (Figure 2A). Cell growth was examined by cell number counting at different time points after andrographolide treatment. Andrographolide treatment for 24, 48, and 72 h obviously decreased HUVEC-C numbers (Figure 2B). We also performed real-time PCR to evaluate cell cycle regulation genes, and observed that andrographolide treatment observably enhanced cell cycle negative regulated genes, including P14arf, P19arf, PTEN, and P53 (Figure 2C). Cell cycle was also determined by flow cytometry; 2 × 10^4^ cells were sorted and our results demonstrated that the majority of cells arrested at the G1 phase and the number arrested at the S phase was decreased after andrographolide treatment (Figure 2D; Supplementary Figure 5). We further performed BrdU incorporation assay, and found that andrographolide treatment significantly inhibited BrdU positive cells (Figures 2E,F). Similar results were obtained from *in vivo* studies. Immunohistochemistry staining against Ki67 and PCNA was performed on slides at E18.5, showing that Ki67 and PCNA positive cells of the hyaloid vascular were dramatically decreased (Figures 2G,H; Supplementary Figures 6–8). These data demonstrated that andrographolide obviously suppressed HUVEC-C proliferation.

### Andrographolide suppresses endothelial cell migration

**Figure 3:**
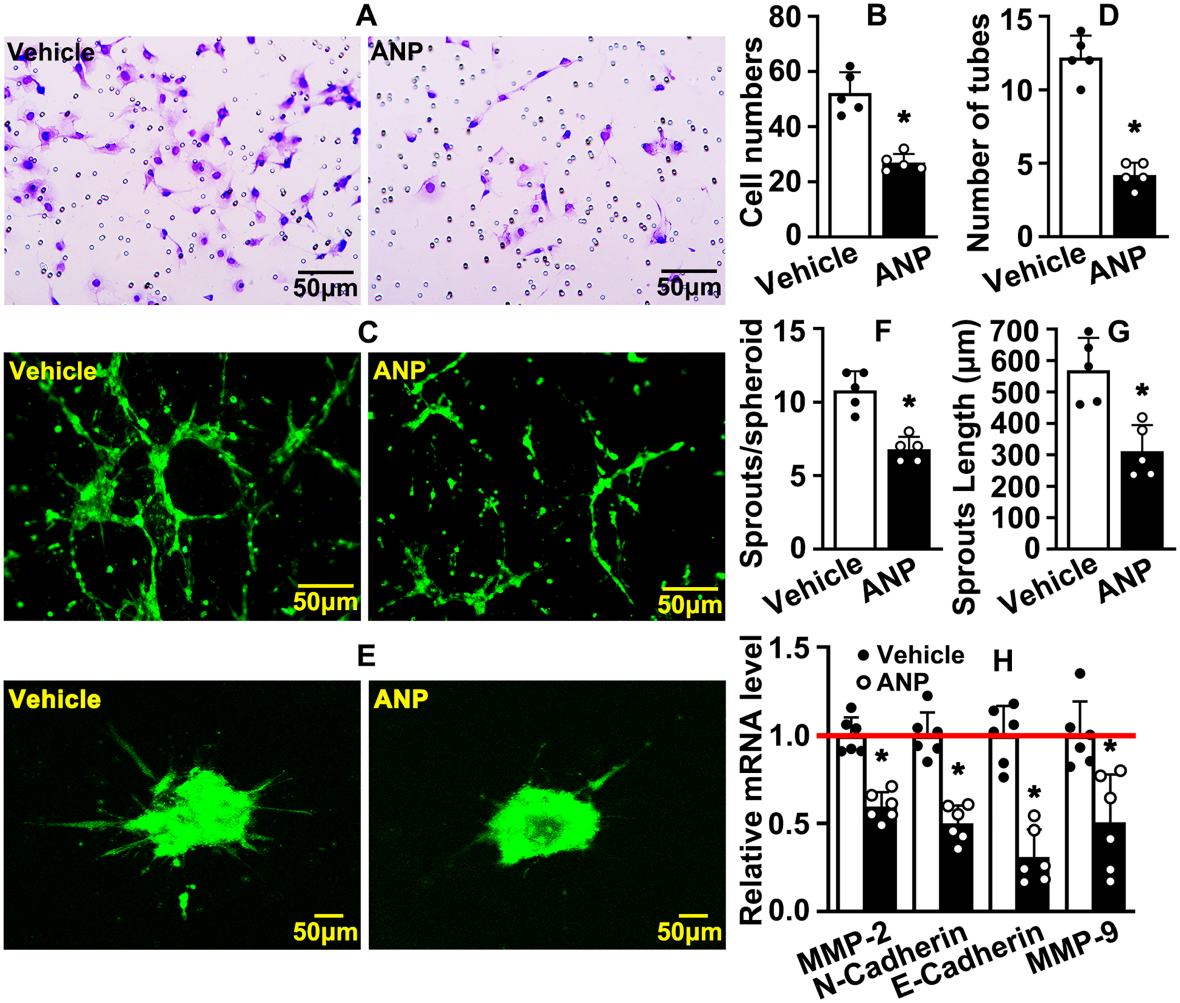
Andrographolide suppresses endothelial cell migration. **(A)** After 12 h of andrographolide treatment, migration of HUVEC-C was detected by Boyden chamber assay (*n* = 5), and migrated cells were quantified in **(B)** (*n* = 5). **(C)** Matrigel-based tube formation assay was performed following andrographolide treatment for 48 h (*n* = 5). The tube numbers were quantified in **(D)** (*n* = 5). **(E)** The migrating ability of HUVEC-C after andrographolide treatment for 24 h was evaluated by spheroidal sprouting assay following Calcein AM staining, and sprouting numbers and sprouting lengths were quantified in **(F)** and **(G)** (*n* = 5). **(H)** HUVEC-C was treated with andrographolide for 30 h and Real-time PCR was performed to evaluate transcription levels of migrative-related genes (*n* = 6). Quantitative data presented as mean ± SEM, *p < 0.05.

The migration of endothelial cells is another hallmark during angiogenesis. We sought to determine whether andrographolide regulated migration of HUVEC-C. HUVEC-C was treated with 5 μM andrographolide and Boyden chamber migration assay was performed. Andrographolide treatment dramatically inhibited HUVEC-C migration, and the number of migrated HUVEC-C was significantly decreased (Figures 3A,B). Next, we performed a Matrigel-based tube formation assay to evaluate whether andrographolide suppressed angiogenesis *in vitro*. Migrating HUVEC-C touching each other and tubes were well formed in vehicle groups. However, following andrographolide treatment, migrating HUVEC-C partially touched each other, and the majority of HUVEC-C were exhibited sporadically, with formed tubes conspicuously inhibited (Figures 3C,D). Similar results were exhibited in our spheroid sprouting assay. Andrographolide treatment obviously suppressed HUVEC-C sprouting, characterized by decreasing sprouting numbers (Figures 3E–G). We further performed real-time PCR to determine the extracellular matrix deposition which is pivotal for maturation of neovascularization. We found that andrographolide treatment dramatically inhibited MMP-2, MMP-9, N-cadherin, and E-cadherin expression (Figure 3H). Our data indicated that andrographolide suppressed HUVEC-C migration.

### The expression of HIF-1a was enhanced during embryonic stage

**Figure 4:**
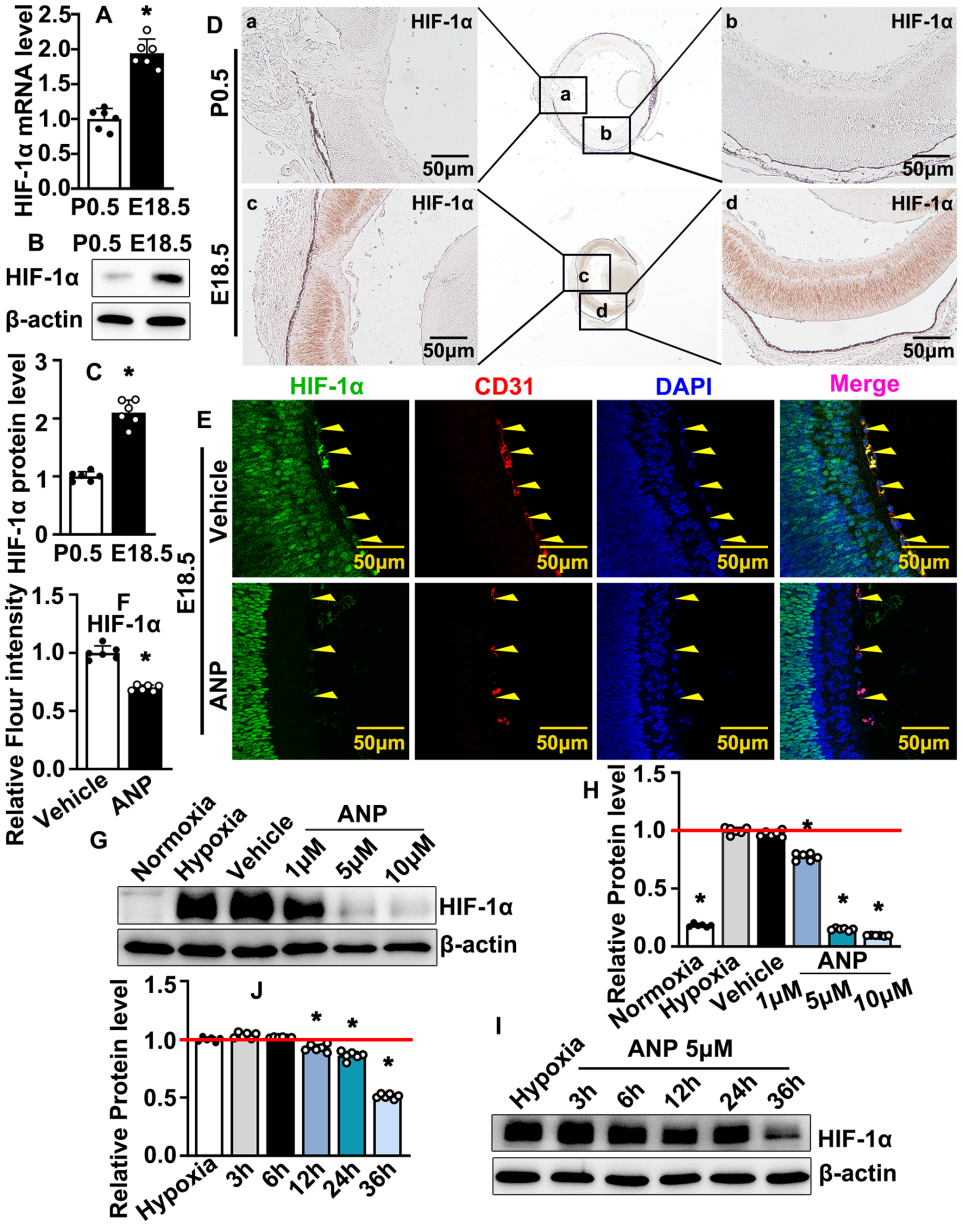
Andrographolide inhibits embryonic hyaloid vascular endothelial HIF-1a expression during murine hyaloid vasculature development. **(A)** Total RNA prepared from retinas at E18.5 and P0. Real-time PCR was performed to evaluate HIF-1a mRNA transcription levels at E18.5 and P0.5 (*n* = 6). **(B)** Total protein prepared from retinas at E18.5 and P0.5. HIF-1a protein levels were determined by western blotting. Relative expression level of HIF-1a was exhibited in **(C)** (*n* = 6). **(D)** IHC staining against HIF-1a antibody performed on slides from P0.5 and E18.5 to determine expression of HIF-1a. Whole images were exhibited in the middle sites and the high resolution images of the corresponding areas were displayed on both sides. **(E)** Immunofluorescence staining against HIF-1a and CD31 antibodies were performed to evaluate HIF1a expression in hyaloid vascular endothelial at E18.5 following andrographolide (10 mg/kg) consecutive treatment. **(F)** Relative expression of HIF-1a in hyaloid vascular endothelial at E18.5 following andrographolide treatment was quantified (*n* = 6). **(G)** Hypoxia conditions were induced in HUVEC-C by 200 μM CoCl_2_ treatment, following different doses of andrographolide treatment (1 μM, 5 μM and 10 μM). The expression levels of HIF-1a were determined by western blotting. **(H)** The quantification of relative HIF-1a protein levels following different doses of andrographolide treatment (*n* = 6). **(I)** Hypoxia conditions were induced in HUVEC-C by 200 μM CoCl_2_ treatment. HUVEC-C was treated with 5 μM andrographolide. Protein was harvested at different time points (3 h, 6 h, 12 h, 24 h, and 36 h) and western blotting was performed to determine HIF-1a expression. **(J)** The quantification of relative HIF-1a expression following andrographolide treated for different time points (*n* = 6). Quantitative data are presented as mean ± SEM, *p < 0.05.

Embryonic blood circulation depends on maternal placental blood supply. Whether hypoxic conditions are exhibited at the embryonic stage still needs to be defined. We collected retinas at E18.5 and postnatal stage 0.5 (P0.5). Real-time PCR was performed to evaluate the transcription levels of the hypoxia-inducible factor (HIF) family. The transcription levels of HIF-1a, HIF-2a, and HIF-3a were enhanced at the embryonic stage (Figure 4A; Supplementary Figure 9A). However, the highest transcription level within the retina was HIF-1a (Supplementary Figure 9B). We sought to determine whether HIF-1a protein could be induced during the embryonic stage. Our western blotting data indicated that higher expression levels of HIF-1a were exhibited at E18.5 compared to P0.5 (Figures 4B,C). We further performed IHC staining against HIF-1a antibodies on slides from E18.5 and P0.5. The expression levels of HIF-1a were almost undetectable at P0.5. However, a high expression level of HIF-1a was exhibited at E18.5, and the highest expression of HIF-1a was located within the retinal neural cells (Figure 4D). These data indicated that, compared to the postnatal stage, a hypoxic condition was exhibited at the embryonic stage.

### Andrographolide inhibits embryonic hyaloid vascular endothelial HIF-1a expression during murine hyaloid vasculature development

Whether the regulation of andrographolide-suppressed hyaloid vasculature development was associated with oxygen supplements is largely unknown. Hypoxia triggers the expression of hypoxia-inducible transcription factors (HIF), and hypoxia conditions contribute to the stabilization of HIF-1, which is essential for vasculogenesis. We performed IF staining against HIF-1a to evaluate the HIF-1a expression at E18.5. Andrographolide treatment dramatically inhibited HIF-1a expression within hyaloid vascular endothelial cells, which was visualized by CD31 staining (Figures 4E,F). That andrographolide could inhibit hyaloid vascular endothelial cells HIF-1a expression at the embryonic stage was also confirmed by IHC staining against HIF-1a antibodies (Supplementary Figure 10). We next induced hypoxia conditions in cultured HUVEC-C by 200 μM CoCl_2_ treatment following different doses of andrographolide treatment (1, 5, 10 μM). Our western blotting results indicated that both 5 μM and 10 μM andrographolide treatment dramatically inhibited HIF-1a expression (Figures 4G,H). We further evaluated HIF-1a expression following different time points of andrographolide treatment (3, 6, 12, 24, and 36 h). Western blotting results indicated that HIF-1a expression decreased after andrographolide treatment for 12 h (Figures 4I,J). These data indicated that andrographolide treatment attenuated the expression of HIF-1a during embryonic hyaloid vasculature development.

### Andrographolide inhibits embryonic hyaloid vascular endothelial VEGFR2 expression during murine hyaloid vasculature development

**Figure 5:**
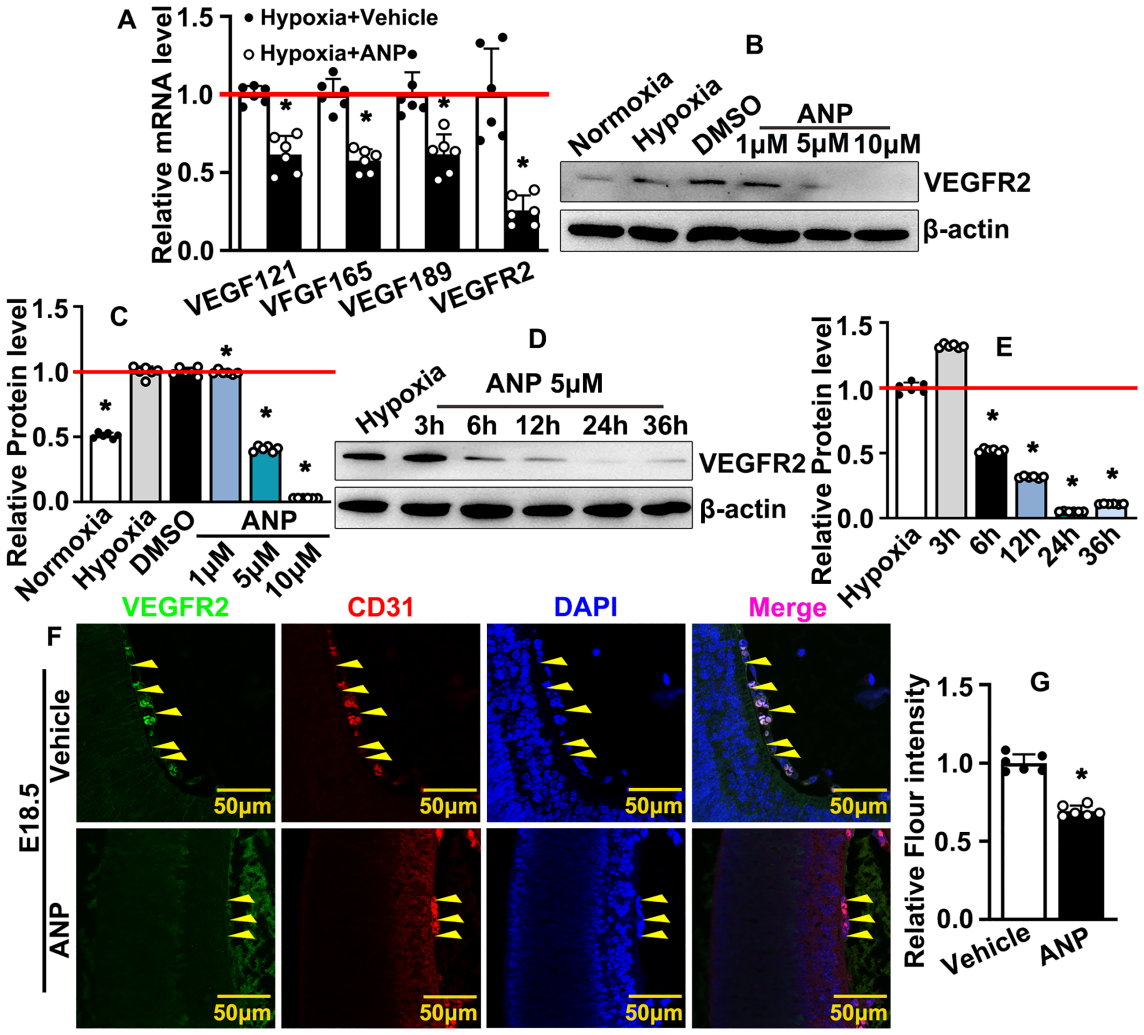
Andrographolide inhibits embryonic hyaloid vascular endothelial VEGFR2 expression during murine hyaloid vasculature development. **(A)** Hypoxia conditions were induced in HUVEC-C by 200 μM CoCl_2_ treatment following andrographolide treatment. Transcription levels of HIFs and VEGFR2 were evaluated by Real-time PCR (*n* = 6). **(B)** Under hypoxia conditions, HUVEC-C was treated with different doses of andrographolide (1 μM, 5 μM and 10 μM); VEGFR2 protein levels were determined by western blotting. **(C)** The quantification of VEGFR2 protein levels in (B) (*n* = 6). **(D)** Hypoxia conditions were induced in HUVEC-C by 200 μM CoCl_2_ treatment, following 5 μM andrographolide treatment. Total protein harvested at 3, 6, 12, 24, and 36 h, and western blotting was performed to determine VEGFR2 expression. **(E)** The quantification of relative VEGFR2 protein levels in **(D)** (*n* = 6). **(F)** Immunofluorescence staining against VEGFR2 and CD31 antibodies was performed to evaluate hyaloid vascular endothelial VEGFR2 expression at E18.5 after andrographolide treatment. **(G)** The quantification of hyaloid vascular endothelial VEGFR2 expression at E18.5 after andrographolide treatment (*n* = 6). Quantitative data presented as mean ± SEM, *p < 0.05.

HIF-1 was reported to induce the transcription of multiple genes. We sought to determine the most essential signaling pathway which was regulated by andrographolide under hypoxia conditions. We treated HUVEC with andrographolide and real-time PCR was performed to evaluate multiple signaling pathways, such as the VEGF, Angiopoietin, Neuropilins, Ephrin, Connexin, Epidermal Growth Factor receptor, and RhoA/Rho-Kinase signaling pathways. Our results indicated that expressions of VEGF121, VEGF165, and VEGF189 were dramatically inhibited after andrographolide treatment, whereas VEGFR1, VEGFR2, and VEGFR3 significantly increased (Supplementary Figure 11). We analyzed the relative expression levels of VEGFRs and found that the highest expression level exhibited in HUVEC after andrographolide treatment was in VEGFR2 (Supplementary Figure 12). To mimic embryonic hypoxic conditions, we treated HUVEC-C with 200 μM CoCl_2_, and hypoxia conditions were confirmed by evaluating the HIF-1a expression using real-time PCR (Supplementary Figure 13). Under hypoxia conditions, andrographolide treatment dramatically suppressed expressions of VEGF121, VEGF165, VEGF189, and VEGFR2 (Figure 5A). Under hypoxia conditions, HUVEC-C was treated with different doses of andrographolide (1 μM, 5 μM, 10 μM) and western blotting was performed to evaluate VEGFR2 expression. We observed that both 5 μM and 10 μM andrographolide treatment significantly suppressed VEGFR2 expression (Figures 5B,C). Under hypoxia conditions, we treated HUVEC-C with 5 μM andrographolide then harvested protein at different time points (3 h, 6 h, 12 h, 24 h, and 36 h). Our western blotting results indicated that andrographolide treatment observably inhibited VEGFR2 expression after 6 h of treatment (Figures 5D,E). We further performed IF staining against VEGFR2 and CD31 antibodies to evaluate VEGFR2 expression in embryonic hyaloid vascular endothelial cells. We observed that andrographolide treatment dramatically suppressed hyaloid vascular endothelial VEGFR2 expression at E18.5 (Figures 5F,G). Our data demonstrated that andrographolide inhibits embryonic hyaloid vascular endothelial VEGFR2 expression during murine hyaloid vasculature development.

### Andrographolide potentially interacts with HIF-1a and VEGFR2 in embryonic hyaloid vascular endothelial cells

**Figure 6:**
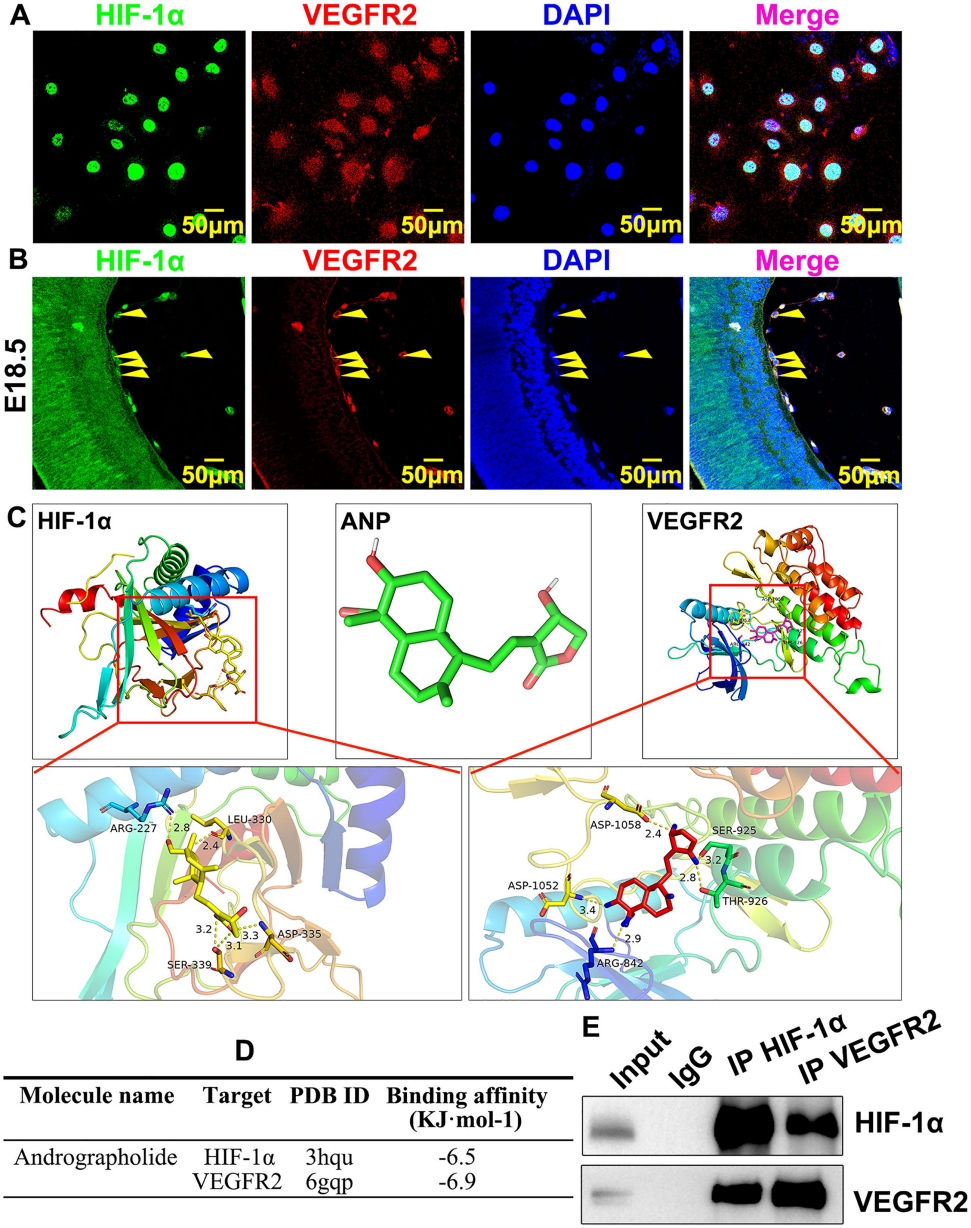
Andrographolide potentially interacts with HIF-1a and VEGFR2 in embryonic hyaloid vascular endothelial cells. **(A)** Hypoxia conditions were induced by 200 μM CoCl_2_ treatment. Immunofluorescence staining against HIF-1a and VEGFR2 antibodies was used to validate co-localization expression of HIF-1a and VEGFR2 in HUVEC-C. **(B)** Immunofluorescence staining against HIF-1a and VEGFR2 antibodies was used to validate co-localization expression of HIF-1a and VEGFR2 in embryonic retinal endothelial at E18.5. **(C)** Molecular docking simulation was performed to evaluate the binding energy of andrographolide with HIF-1a and VEGFR2 using Autodock Vina 1.5.6 software developed by Olson’s research group. Andrographolide worked as receptor, HIF-1a and VEGFR2 were used as ligands to detect the docking sites between receptors and ligands. The three-dimensional structures of HIF-1a and VEGFR2 were obtained from the RCSBPDB database (http://www.rcsb.org/). When a binding energy value was less than zero, those proteins were considered spontaneously binding and interacting with each other. **(D)** The binding energy of andrographolide with HIF-1a and VEGFR2 was based on Molecular docking simulation. **(E)** Hypoxia conditions were induced in HUVEC by 200 μM CoCl_2_ treatment. The interaction between HIF-1a and VEGFR2 was validated by co-immunoprecipitation.

How andrographolide regulates HIF-1a and VEGFR2 during murine hyaloid vasculature development is largely unknown. We induced hypoxia conditions in HUVEC-C by CoCl_2_ treatment to trigger HIF-1a expression, and IF staining against HIF-1a and VEGFR2 antibodies was performed to determine whether there was co-localization expression of HIF-1a and VEGFR2 in HUVEC-C. We observed that the majority of HIF-1a was strictly expressed within the nucleus, whereas VEGFR2 was ubiquitously expressed within HUVEC-C; HIF-1a and VEGFR2 co-localization was expressed in the nucleus (Figure 6A). We also performed IF staining to confirm co-localization expression of HIF-1a and VEGFR2 in hyaloid vascular endothelial cells. We observed that HIF-1a and VEGFR2 co-localization was expressed in hyaloid vascular endothelial cells at E18.5 (Figure 6B). We further sought to analyze the molecular docking simulation of andrographolide with HIF-1a and VEGFR2, which was determined by the binding energy of andrographolide with HIF-1a and VEGFR2 using Autodock Vina 1.5.6 software developed by Olson’s research group. The three-dimensional structures of HIF-1a and VEGFR2 were obtained from the RCSBPDB database (http://www.rcsb.org/). Andrographolide worked as a receptor, and HIF-1a and VEGFR2 were used as ligands to detect the docking sites between receptors and ligands. Multiple potential binding areas of andrographolide with HIF-1a and VEGFR2 were exhibited (Figure 6C). When a binding energy value was less than zero, those proteins were considered spontaneously binding and interacting with each other. It was observed that −6.5 of binding energy was exhibited between andrographolide with HIF-1a, and − 6.9 of binding energy was exhibited between andrographolide with VEGFR2 (Figure 6D). We further performed co-immunoprecipitation to determine whether HIF-1a interacted with VEGFR2 *in vitro*. Total protein from HUVEC was prepared using RIPA buffer after CoCl_2_ treatment and the co-immunoprecipitated targets were evaluated using western blotting. Our result indicated that HIF-1a and VEGFR2 could interact with each other *in vitro* (Figure 6E). Our data demonstrated that andrographolide at least in part interacts with HIF-1a and VEGFR2 in embryonic hyaloid vascular endothelial cells.

### Interruption VEGF receptors further suppresses andrographolide mediated embryonic hyaloid vasculature development

**Figure 7:**
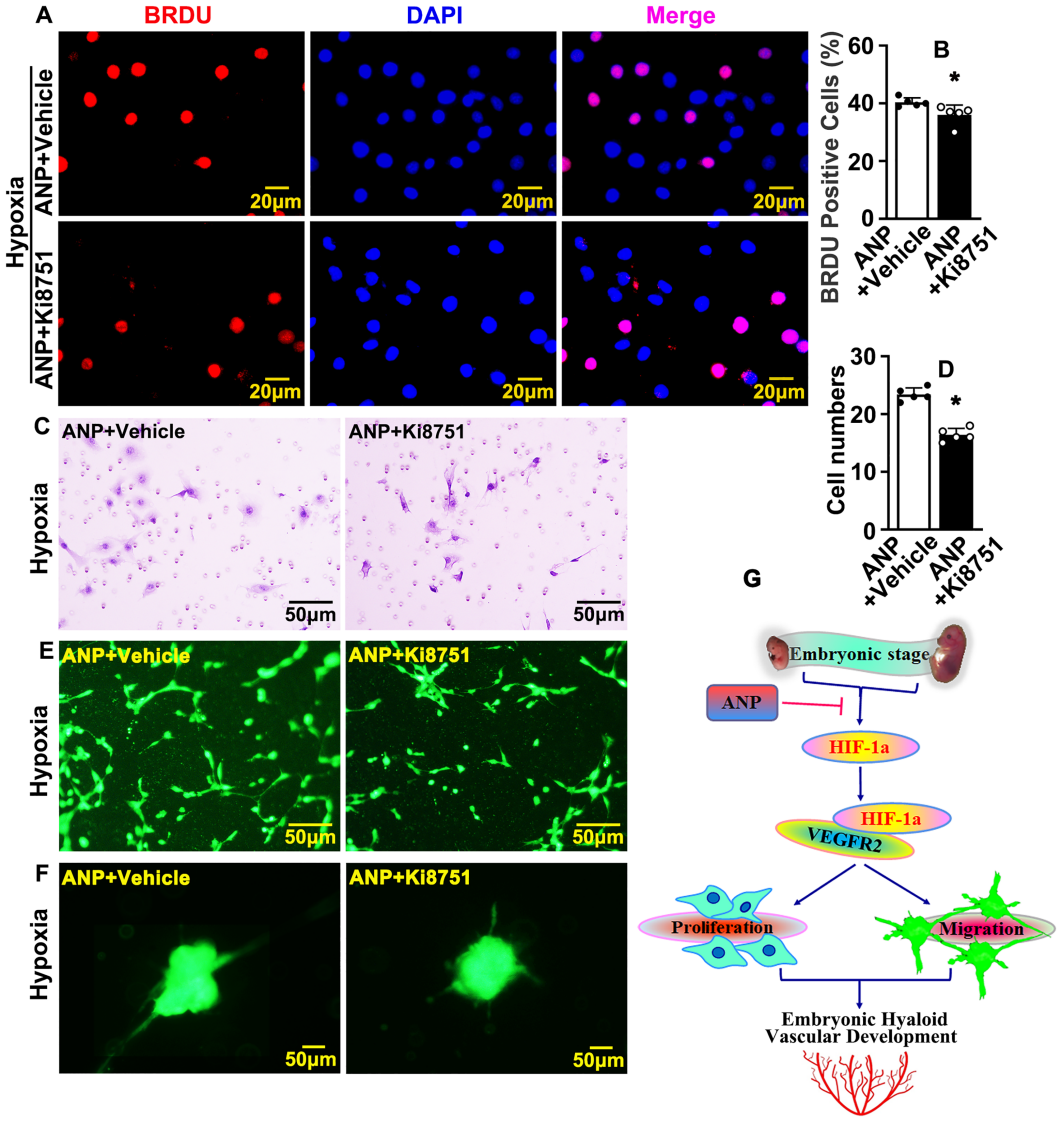
Interruption VEGF receptors further suppresses andrographolide mediated embryonic hyaloid vasculature development. **(A)** Hypoxia conditions were induced in HUVEC-C by 200 μM CoCl_2_ treatment and VEGFR2 inhibition performed by Ki8751 treatment (Ki8751, a VEGFR2 inhibitor). Following BrdU labeling reagent labeled for 24 h after treatment of andrographolide. Immunofluorescence staining was performed to determine BrdU incorporation. **(B)** The quantification of BrdU positive cells from **(A)** (*n* = 5). **(C)** Boyden chamber cell migration assay was performed to validate the role of VEGFR2 on cell migration after VEGFR2 inhibition following andrographolide under hypoxia conditions. **(D)** The quantification of migrated HUVEC-C from **(C)** (*n* = 5). **(E)** Matrigel-based tube formation assay was performed to validate the role of VEGFR2 on tube formation after VEGFR2 inhibition with andrographolide treatment under hypoxia conditions. **(F)** Spheroid sprouting assay was performed to validate the role of VEGFR2 after VEGFR2 inhibition with andrographolide treatment under hypoxia conditions. **(G)** The schematic diagram indicates how andrographolide regulates embryonic hyaloid vascular development.

In order to determine whether andrographolide regulated embryonic hyaloid vasculature development primarily through HIF-1a/VEGF signaling pathways, we treated HUVEC-C with Ki8751, a VEGFR2 inhibitor, to suppress the viability of VEGFR2. Firstly, we found that Ki8751 obviously suppressed BrdU incorporation when tested using BrdU incorporation assay, and the viability of HUVEC-C was determined by Cell Counting Kit-8 assay (Supplementary Figures 14A–C). We next performed BrdU incorporation assay in HUVEC-C with andrographolide with or without Ki8751 treatment. Our results indicated that andrographolide inhibited BrdU incorporation, and the inhibition effect of andrographolide was further suppressed after Ki8751 treatment (Figures 7A,B). Next, we performed Boyden chamber migration assay to detect cell migration ability when treated with Ki8751. The result showed that Ki8751 treatment also suppressed HUVEC-C migration (Supplementary Figures 15A,B). We then detected the cell migration ability of HUVEC-C with andrographolide with or without Ki8751 treatment. Our data demonstrated that andrographolide inhibited cell migration ability, and the inhibiting effect of andrographolide was also further suppressed after Ki8751 treatment (Figures 7C,D). Meanwhile, the inhibiting effect on tube formation and endothelial cell sprouting treated with andrographolide were also further suppressed after Ki8751 treatment (Figures 7E,F; Supplementary Figures 16, 17).

In summary, our data demonstrated that hypoxia conditions trigger HIF-1a expression. HIF-1a actives the VEGF signaling pathway through interacting with VEGFR2, resulting in promoting the proliferation and migration of hyaloid vascular endothelial cells, and eventually regulating murine hyaloid vasculature development during embryonic retinal development. Andrographolide suppresses HIF-1a and VEGFR2 expression, at least in part, through interacting with HIF-1a and VEGFR2, and results in suppressing embryonic hyaloid vasculature development (Figure 7G).

## Discussion

This study provides evidence of andrographolide-suppressed embryonic hyaloid vasculature development through HIF-1a/VEGFR2 signaling pathways. We performed real-time PCR to screen the potential signaling pathways involved following andrographolide treatment. We found that the expression of VEGF121, VEGF165, and VEGF189 were inhibited, while the expression of VEGFR1, VEGFR2, and VEGFR3 were significantly increased under normoxia conditions (Supplementary Figure 11). This was probably due to the negative feedback regulation mechanism that increased VEGF receptors and may indicate a deficiency of VEGF (40). We verified that hypoxic conditions were exhibited at the embryonic stage. We later used CoCl2 to induce hypoxia to simulate embryonic hypoxic conditions. Under hypoxia conditions, our results demonstrated that andrographolide treatment dramatically suppressed expressions of VEGF121, VEGF165, VEGF189, and VEGFR2 (Figure 5A). This may be because andrographolide inhibits the expression of HIF-1a, thereby inhibiting the expression of its downstream molecules. But how andrographolide treatment increased VEGFR2 expression under normoxia conditions needs further study. Hypoxia conditions were exhibited within hyaloid vascular endothelial cells during the embryonic stage. Hypoxia conditions triggered HIF-1a expression, and HIF-1a activated VEGF signaling pathways through interacting with VEGFR2, resulting in the regulation of murine hyaloid vasculature development during embryonic hyaloid development. Our study demonstrated that andrographolide suppressed HIF-1a and VEGFR2 expression and, at least in part, interrupted the interaction between HIF-1a and VEGFR2, resulting in suppressing endothelial cell proliferation, migration, and eventually suppressing murine hyaloid vasculature development. Hypoxia stabilized HIF-1a, and HIF-1a activated VEGF expression and its receptors, VEGFR2. But in normoxic conditions, a prolyl hydroxylase dehydroxylates HIF-1a resulted in ubiquitination and degradation. VEGFR2 is reported to be internalized through clathrin-dependent and -independent routes or Macropinocytosis. But how the loss of interaction between VEGFR2 and HIF-1a could lead to their common degradation needs further study.

Retinal vascular development undergoes tremendous morphological changes and vascular remodeling. Vasculogenesis and angiogenesis are two typical processes by which new blood vessels are formed (9, 41). Vasculogenesis is defined as the formation of a primitive vascular network from the differentiation of angioblasts into endothelial cells, whereas angiogenesis refers to the growth of new capillaries from pre-existing blood vessels (41). Embryonic retinal vascular vessels are formed by both vasculogenesis and angiogenesis. In this study, we treated pregnant C57/BL6 mice with consecutive intraperitoneal injections of andrographolide, and aimed to determine whether and how andrographolide regulated normal embryonic hyaloid vasculature development. However, our studies could not distinguish between vasculogenesis and angiogenesis. The proliferation and migration of endothelial cells plays a pivotal role during retinal vascular development. In this study, we focused on investigating the role of andrographolide on the proliferation and migration of endothelial cells. However, multiple cell types also contribute to embryonic retinal vascular development. Astrocytes form a scaffold and promote newly formed blood vessels during retinal development (42). Retinal microglia are essential for the formation of subretinal neovascular vessels (43). In this study, we demonstrated that hypoxia conditions were exhibited within embryonic retinas, whereas other stresses were also involved during retinal vascular development. Environmental oxygen controls the proliferation of astrocytes which are critical in the regulation of retinal angiogenesis (44). Disturbed blood flow related shear stress is also involved in regulating embryonic retinal angiogenesis (45). The integrity of the retinal vascular barrier contributes to embryonic retinal vascular development (46, 47). Zebrafish embryos are an innovative model for studying retinal angiogenesis and can be employed for studying angiogenesis-dependent eye diseases (48).

*Andrographis paniculata* is traditionally used in the treatment of diseases with burning symptoms due to its cooling properties. Andrographolide is a key principle isolated from *Andrographis paniculate* (30). Our previous publication demonstrated that andrographolide played a role in both maintaining gastric vascular homeostasis and regulating pathological vascular remodeling (30, 31). In this study, we demonstrated that andrographolide regulated normal embryonic hyaloid vasculature development. These data not only advances knowledge of the mechanisms of hyaloid vasculature development, but may also potentially be employed clinically for the prevention and treatment of other vascular diseases.

## Data availability statement

The original contributions presented in the study are included in the article/supplementary material, further inquiries can be directed to the corresponding author.

## Ethics statement

The animal study was reviewed and approved by the Experimental Animal Ethics Committee of Chengdu University of Traditional Chinese Medicine.

## Author contributions

YW, LY, and LS contributed to the research design. XW, ZW, QG, ZJ, WH, JL, HL, SZ, and HZ contributed to the experiments. ZC, YW, and HX contributed data analysis. YW and ZJ contributed to manuscript writing and revising. All authors contributed to the article and approved the submitted version.

## Funding

This study was supported by a grant (81741007 and 81870363) from the National Natural Science Foundation of China, grant 2020JDTD0025 from Science & Technology Departments of Sichuan province, grant 008066, 030038199, BJRC2018001, JSZX2018004, 030041224, ZKYY2004/030055180, 242030016, MPRC2021038, and CCCXFH202205 from Chengdu University of Traditional Chinese Medicine.

## Conflict of interest

The authors declare that the research was conducted in the absence of any commercial or financial relationships that could be construed as a potential conflict of interest.

## Publisher’s note

All claims expressed in this article are solely those of the authors and do not necessarily represent those of their affiliated organizations, or those of the publisher, the editors and the reviewers. Any product that may be evaluated in this article, or claim that may be made by its manufacturer, is not guaranteed or endorsed by the publisher.
