# Supplementary material for: Andrographolide suppresses hypoxia-induced embryonic hyaloid vascular system development through HIF-1a/VEGFR2 signaling pathway
Source: Front Cardiovasc Med. 2023 Feb 8;10:1090938. doi: 10.3389/fcvm.2023.1090938 (PMC9944699; doi:10.3389/fcvm.2023.1090938)
Supplement: Supplementary file 1 [file Data_Sheet_1.pdf]

Supplementary Figure 1

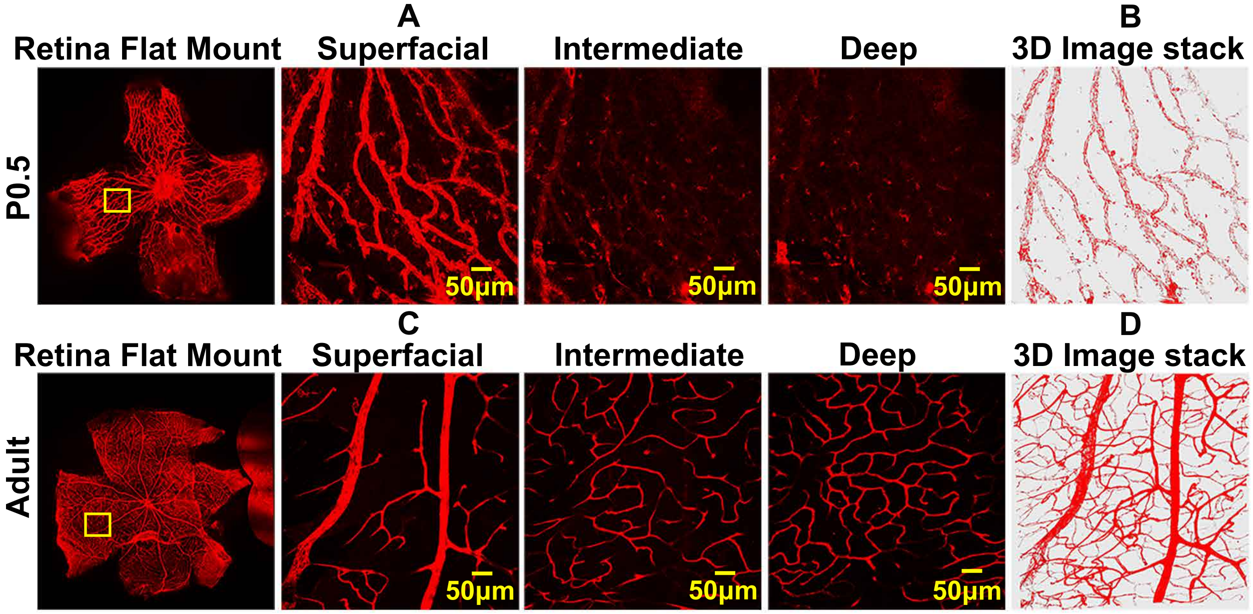

Supplementary Figure 2

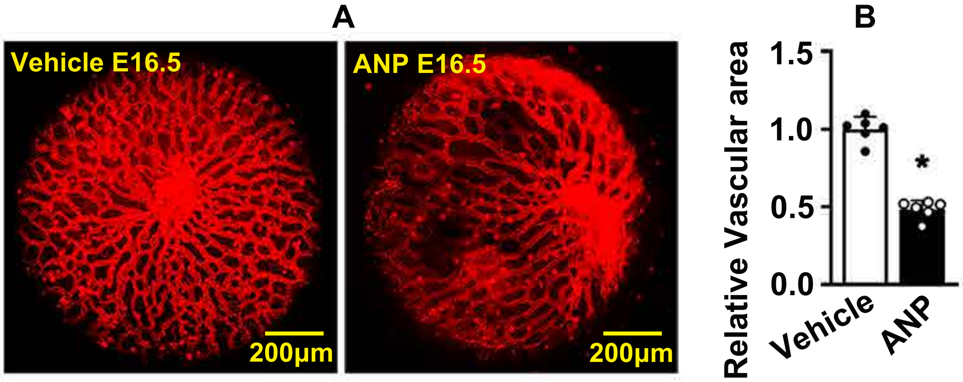

Supplementary Figure 3

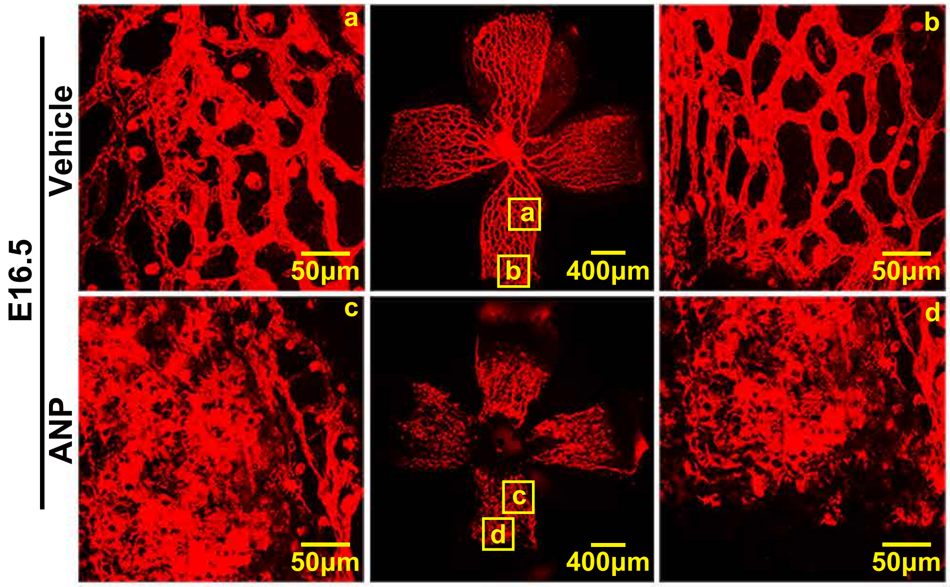

Supplementary Figure 4

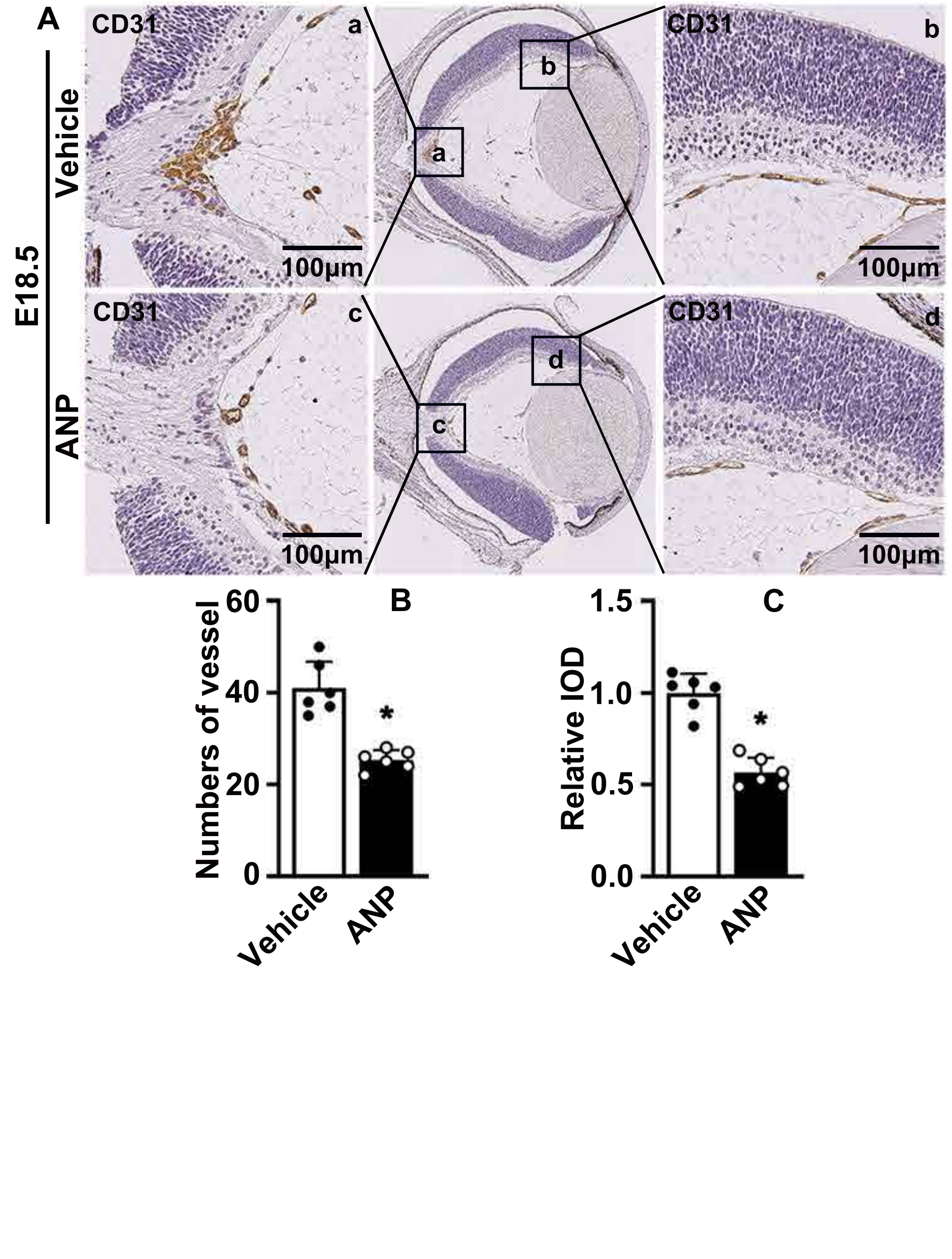

Supplementary Figure 5

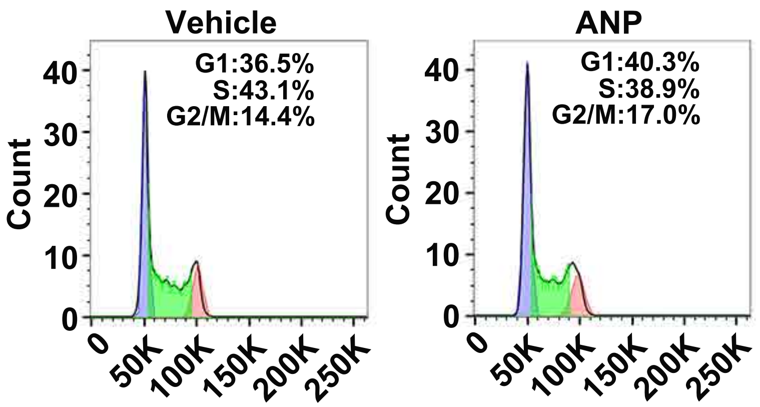

Supplementary Figure 6

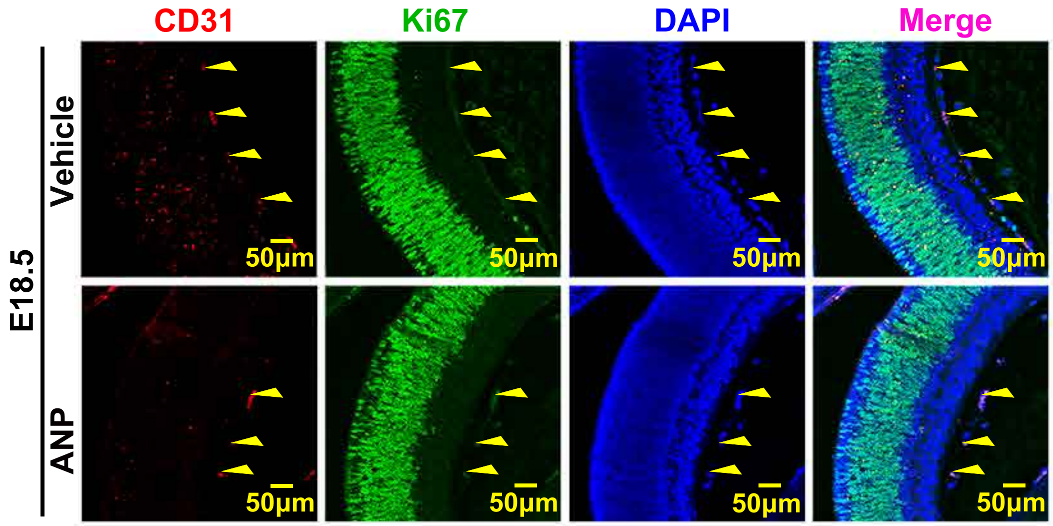

Supplementary Figure 7

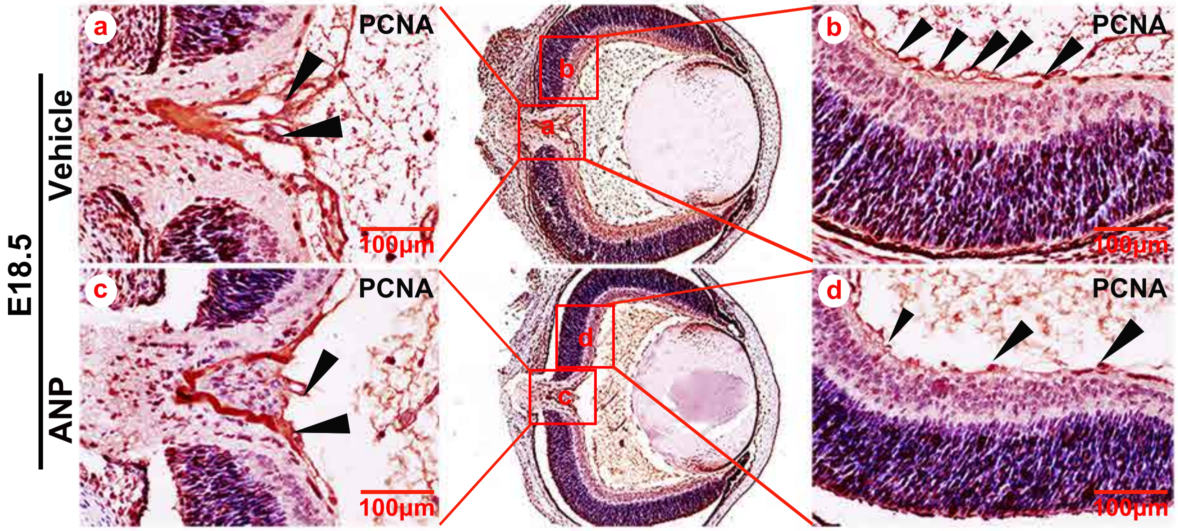

Supplementary Figure 8

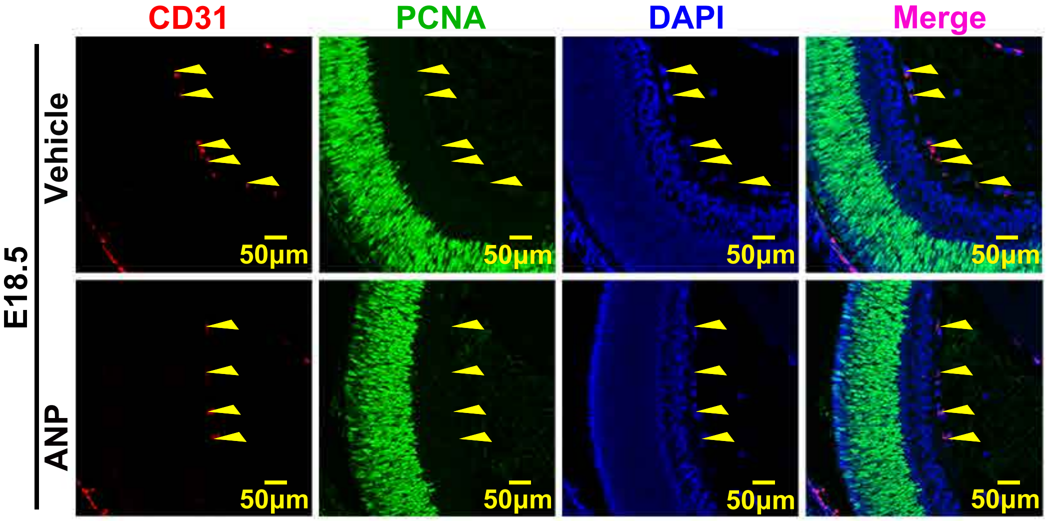

Supplementary Figure 9

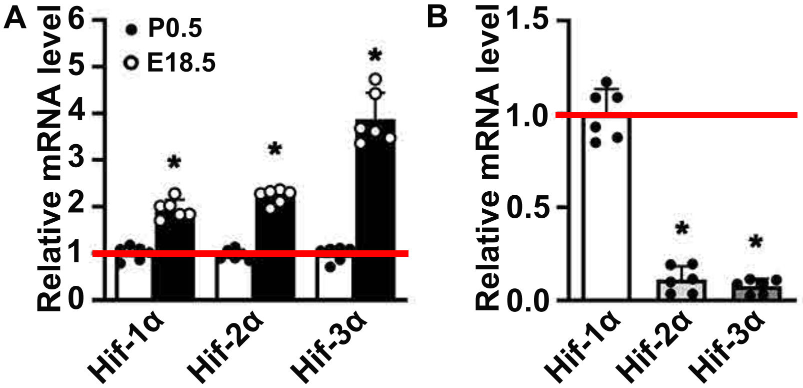

Supplementary Figure 10

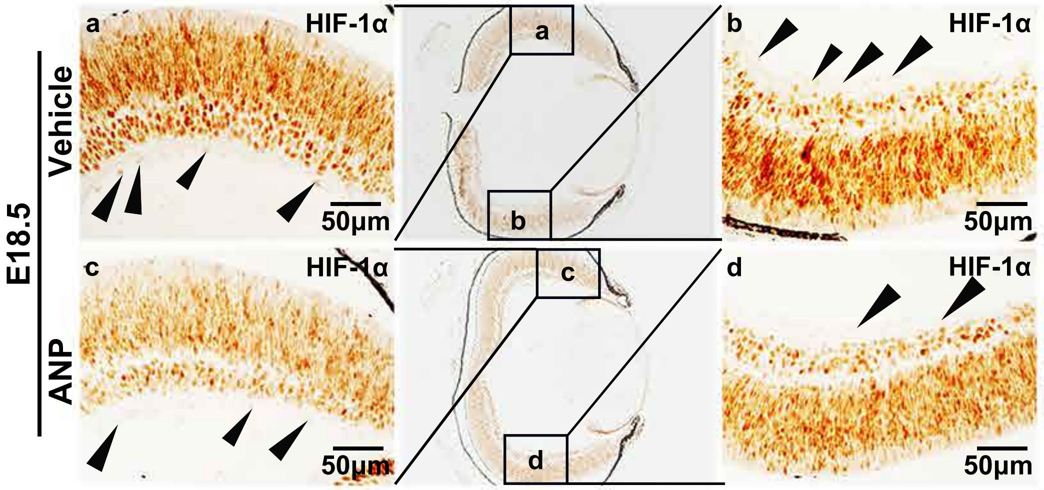

Supplementary Figure 11

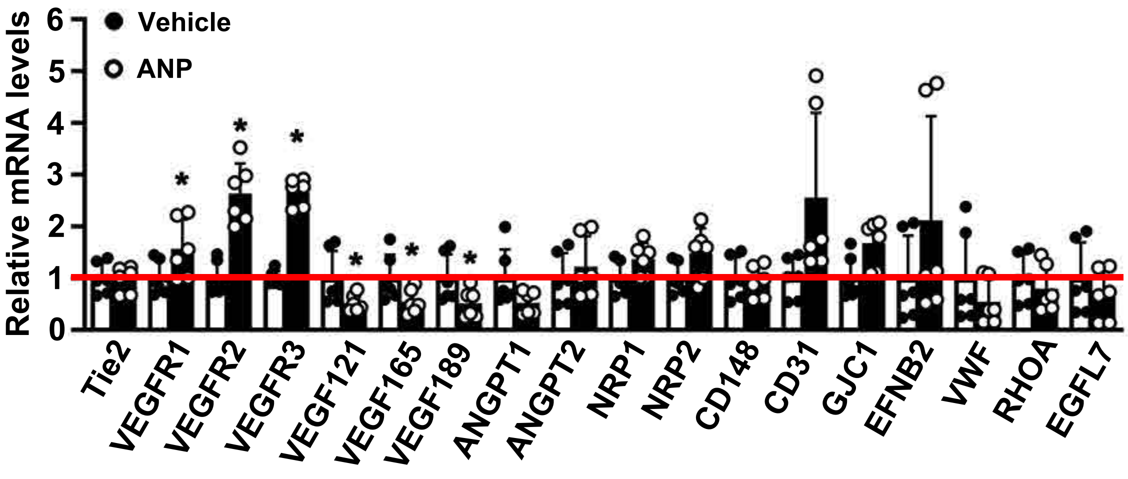

Supplementary Figure 12

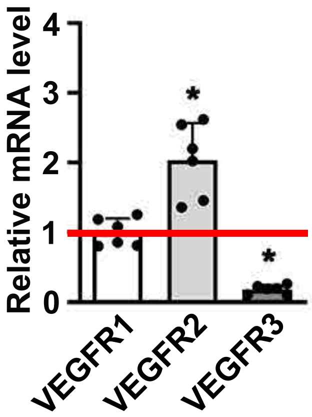

Supplementary Figure 13

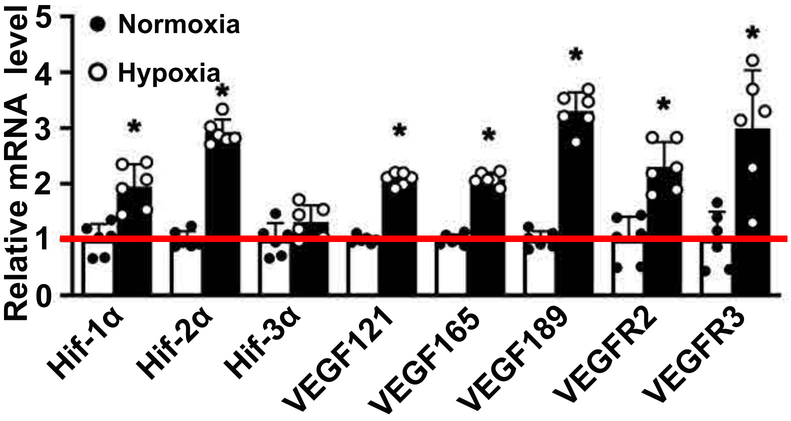

Supplementary Figure 14

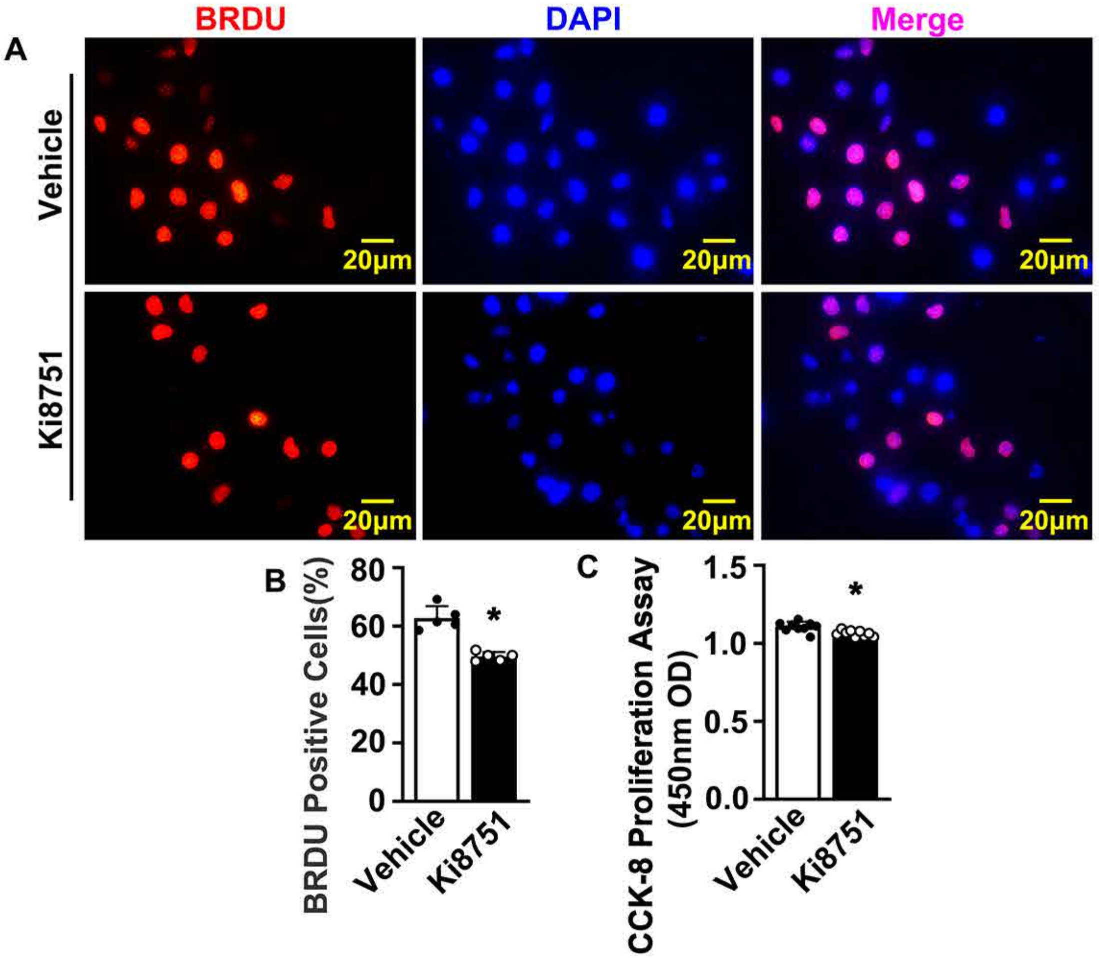

Supplementary Figure 15

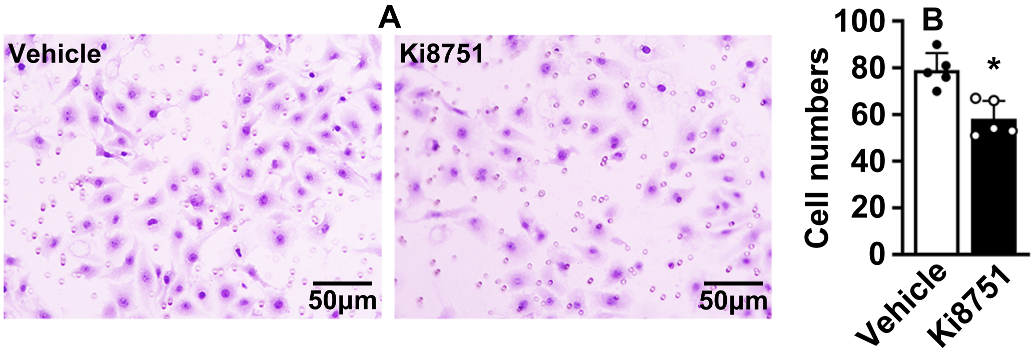

Supplementary Figure 16

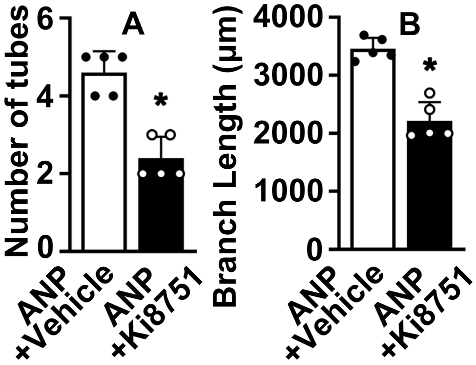

Supplementary Figure 17

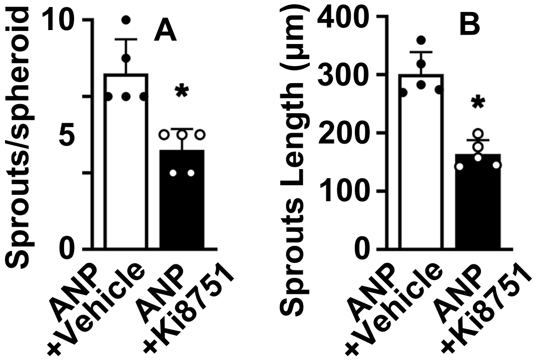

Supplementary Figure 18. List of primer sequences used for Real Time PCR

Gene Name	*Species*	*Sequence*
VEGF121	Human	F:5'-CCCTGATGAGATCGAGTACATCTT-3'
VEGF121	Human	R:5'-GCCTCGGCTTGTCACATTTT-3'
VEGF165	Human	F:5'-CCCTGATGAGATCGAGTACATCTT-3'
VEGF165	Human	R:5'-AGCAAGGCCCACAGGGATTT-3'
VEGF189	Human	F:5'-CCCTGATGAGATCGAGTACATCTT-3'
VEGF189	Human	R:5'-AACGCTCCAGGACTTATACCG-3'
VEGFR1	Human	F:5'-TCTCACACATCGACAAACCAATACA-3'
VEGFR1	Human	R:5'-GGTAGCAGTACAATTGAGGACAAGA-3'
VEGFR2	Human	F:5'-GCAGGGGACAGAGGGACTTG-3'
VEGFR2	Human	R:5'-GAGGCCATCGCTGCACTCA-3'
VEGFR3	Human	F:5'-GACAGCTACAAGTACGAGCATCTG-3'
VEGFR3	Human	R:5'-CGTTCTTGCAGTCGAGCAGAA-3'
CD31	Human	F:5'-TTAGCCTGAGGAATTGCTGTGTT-3'
CD31	Human	R:5'-AGCTGTAGGAGGAGAATCCATC-3'
CD148	Human	F:5'-AGTACACACGGCCCAGCAAT-3'
CD148	Human	R:5'-GAGGCGTCATCAAAGTTCTGC-3'
Tie2	Human	F:5'-TTGAAGTGGAGAGAAGGTCTG-3'
Tie2	Human	R:5'-GTTGACTCTAGCTCGGACCAC-3'
ANGPT1	Human	F:5'-AACATGGGCAATGTGCCTACACTT-3'
ANGPT1	Human	R:5'-CATTCTGCTGTATCTGGGCCATCT-3'
ANGPT2	Human	F:5'-CAGATTTTGGACCAGACCAGTGA-3'
ANGPT2	Human	R:5'-TCAATGATGGAATTTTGCCTTGGA-3'
NRP1	Human	F:5'-CAGAAAAGCCCACGGTCAT-3'
NRP1	Human	R:5'-CAGCCAAATTCACAGTTAAAACC-3'
NRP2	Human	F:5'-AAGTCTCCTACAGCCTAAACGG-3'
NRP2	Human	R:5'-GATGTCAGGGGTGTCATAGTGC-3'
GJC1	Human	F:5'-AGCTGTAGGAAGGAGAATCCATC-3'
GJC1	Human	R:5'-TGCAAACGCATCATAACAGACA-3'
EFNB2	Human	F:5'-TTCGACAACAAGTCCCTTTG-3'
EFNB2	Human	R:5'-GATGTTGTTCCCCGAATGTC-3'
VWF	Human	F:5'-GTCGAGCTGCACAGTGACATG-3'
VWF	Human	R:5'-GCACCATAAACGTTGACTTCCA-3'
RHOA	Human	F:5'-GAAGAGGCTGGACTCGGATT-3'
RHOA	Human	R:5'-AGCAAGCATGTCTTTCCACA-3'
F3	Human	F:5'-CACTACAAATACTGTGGCAG-3'
F3	Human	R:5'-TCCAATCTCCTGACTTAGTG-3'
EGFL7	Human	F:5'-TGGATGAATGCAGTGCTAGG-3'
EGFL7	Human	R:5'-CCTTGGGCACACAGAGTGTA-3'

Gene Name	*Species*	*Sequence*
HIF-1α	Human	F:5'-AGAGGTTGAGGGACGGAGAT-3'
HIF-1α	Human	R:5'-TCCGACATTGGGAGCTCATT-3'
HIF-2α	Human	F:5'-CCTTCCGACTCCCAGCATTC-3'
HIF-2α	Human	R:5'-GAGGCTGTCAGACCCGAAAA-3'
HIF-3α	Human	F:5'-GGATGGGGCTTCAGTAGCAG-3'
HIF-3α	Human	R:5'-GTTCAGGAGTGGGGTGCTG-3'
HIF-1α	Mouse	F:5'-GCGGCGAGAACGAGAAGAAA-3'
HIF-1α	Mouse	R:5'-GGGGAAGTGGCAACTGATGA-3'
HIF-2α	Mouse	F:5'-GCACCAGCAGTTCACACTTG-3'
HIF-2α	Mouse	R:5'-AATTCATCGGGGGCCATGTT-3'
HIF-3α	Mouse	F:5'-CAGCGCGTGAGGTCGAA-3'
HIF-3α	Mouse	R:5'-ATTGTGAGGCGCATGATGGA-3'
P14arf	Human	F:5'-ATGGTGCGCAGGTTCTTGG-3'
P14arf	Human	R:5'-TGCGGGCATGGTTACTGCCTC-3'
P19arf	Human	F:5'-CCCTCGTGCTGAGCTACTGA-3'
P19arf	Human	R:5'-ACCACCAGCGTGTCCAGGAA-3'
PTEN	Human	F:5'-CCAGTCAGAGGCGCTATGTG-3'
PTEN	Human	R:5'-ACTTGTCTTCCCGTCGTGTG-3'
P53	Human	F:5'-CCACCATCCACTACAACTACAT-3'
P53	Human	R:5'-CAAACACGGACAGGACCC-3'
MMP-2	Human	F:5'-TGGCGATGGATACCCCTTT-3'
MMP-2	Human	R:5'-TTCTCCCAAGGTCCATAGCTCAT-3'
MMP-9	Human	F:5'-CCTGGGCAGATTCCAAACCT-3'
MMP-9	Human	R:5'-GCAAGTCTTCCGAGTAGTTTTGGAT-3'
E-Cadherin	Human	F:5'-CGAGAGCTACACGTTCACGG-3'
E-Cadherin	Human	R:5'-GGGTGTCGAGGGAAAAATAGG-3'
N-Cadherin	Human	F:5'-TTTGATGGAGGTCTCCTAACACC-3'
N-Cadherin	Human	R:5'-ACGTTTAACACGTTGGAAATGTG-3'
